# Walking the Line with Ticagrelor: Meta-Analysis Comparing the Safety and Efficacy of Ticagrelor Monotherapy after a Short Course of Ticagrelor-Based Dual Antiplatelet Therapy versus Standard Therapy in Complex Percutaneous Coronary Intervention

**DOI:** 10.3390/jcm10235506

**Published:** 2021-11-24

**Authors:** Francesco Condello, Matteo Sturla, Riccardo Terzi, Alberto Polimeni, Giulio G. Stefanini

**Affiliations:** 1 Department of Biomedical Sciences, Humanitas University, Pieve Emanuele, 20090 Milan, Italy; fra.condello94@gmail.com (F.C.); matteo.sturla@st.hunimed.eu (M.S.); riccardo.terzi@st.hunimed.eu (R.T.); 2 IRCCS Humanitas Research Hospital, Rozzano, 20089 Milan, Italy; 3 Center for Cardiovascular Diseases, Department of Medical and Surgical Sciences, Division of Cardiology and Research, “Magna Graecia” University, Viale Europa Snc, 88100 Catanzaro, Italy; polimeni@unicz.it

**Keywords:** dual antiplatelet therapy, percutaneous coronary intervention, complex PCI, drug eluting stent, ticagrelor monotherapy

## Abstract

(1) Shorter-duration dual antiplatelet therapy (DAPT) followed by single antiplatelet therapy has been shown to significantly reduce bleeding events while preserving anti-ischemic effects in patients undergoing conventional percutaneous coronary interventions (PCI). Whether this strategy is also safe and effective in complex PCI remains elusive; (2) A systematic search of randomized controlled trials comparing a short course of ticagrelor-based DAPT versus standard DAPT in patients undergoing complex PCI was performed; (3) Of 10,689 studies screened, 3 were identified for a total of 4176 participants on ticagrelor monotherapy after a short course of ticagrelor-based DAPT, and 4209 on standard DAPT. The pooled analysis revealed no difference in the outcomes of major bleeding, myocardial infarction, definite or probable stent thrombosis and ischemic stroke. A significant reduction in the risk of cardiovascular death (incidence rate ratio (IRR) 0.52; 95% CI 0.28–0.96; *p* = 0.04), all-cause death (IRR 0.65; 95% CI 0.49–0.86; *p* = 0.003), and any bleeding events (IRR 0.62; 95% CI 0.47–0.81; *p* < 0.001) was seen in the shorter DAPT group; (4) Among patients undergoing complex PCI, ticagrelor monotherapy after a short course of ticagrelor-based DAPT significantly reduced bleeding risk without increasing ischemic risk. More data are needed to definitively explain mortality benefits.

## 1. Introduction

Current guidelines recommend dual antiplatelet therapy (DAPT) with aspirin and clopidogrel for 6 months in patients with stable coronary artery disease (CAD) after percutaneous coronary intervention (PCI). Instead, a duration of 12 months is recommended in patients presenting with acute coronary syndrome (ACS), with a more potent P2Y12 inhibitor such as ticagrelor or prasugrel, on top of aspirin [1]. Patients with more advanced CAD and requiring complex PCI for revascularization are at an increased risk for adverse ischemic events [2]. Nevertheless, prolonged DAPT regimens to combat ischemic risk are inherently associated with increased bleeding risk, in itself known to be associated with an increased risk of morbidity and mortality [2]. Multiple risk scores are available to assess patient ischemic and/or bleeding risk (PARIS, ACUITY, CRUSADE, ARC-HBR, Blee-MACS risk scores) [3], but only two are currently recommended by the European Society of Cardiology (ESC) to guide and inform decision making on DAPT duration, with a IIb level of recommendation: the PRECISE-DAPT score and the DAPT score. Nevertheless, none of the aforementioned scores have been prospectively tested in randomized clinical trials (RCT) [1]. Therefore, careful evaluation of clinical and procedural characteristics at an individual patient level is essential in determining the optimal antiplatelet strategy, particularly in patients at high risk for adverse ischemic events.

Ticagrelor monotherapy after shortened ticagrelor-based DAPT strategies of 1 to 3 months has proven to be effective in mitigating the bleeding risks associated with long-term DAPT while maintaining protection against ischemic events in patients undergoing conventional PCI [4,5].

An unsolved question is whether short-duration ticagrelor-based DAPT followed by ticagrelor monotherapy can maintain the delicate balance between ischemic and bleeding risk associated with the clinical and procedural risk factors that define a “complex” PCI procedure. Although no universal definition of complex PCI procedure exists, commonly used criteria were first described by Giustino et al. and include: three vessels treated, ≥three lesions treated, total stent length > 60 mm, bifurcation with two stents implanted, and chronic total occlusion [1,2,6]. Additional features such as the use of any atherectomy device, left main as target vessel, surgical vein bypass graft, and severely calcified lesions have also been considered as additional procedural risk factors of complex PCI [7]. In real-world clinical practice, non-procedural features including clinical patient characteristics (diabetes, chronic kidney disease, ACS at presentation), hemodynamic compromise (heart failure requiring hemodynamic support), and operator experience represent key features of “complex” PCI, driving procedural decisions as well as patient clinical outcomes [8]. 

With sufficient high-quality trial data available surrounding this question, a meta-analysis of RCTs is warranted.

## 2. Materials and Methods

Electronic databases (PubMed, Embase and Cochrane) were searched independently by two reviewers (FC, MS) from inception until 29 July 2021 to identify relevant studies, using the following MeSH terms: “Dual antiplatelet therapy”, “DAPT”, “P2Y12 inhibitor”, “ticagrelor”, “coronary artery disease”, “CAD”, “chronic ischemic heart disease”, “angina”, “acute coronary syndrome”, “myocardial infarction”, and “percutaneous coronary intervention”, “PCI”, “angioplasty”, “drug eluting stent”, “DES”. Studies were included if they: (1) were published in a peer review journal; (2) were RCTs; (3) included patients undergoing complex PCI with drug-eluting stent (DES) implantation; and (4) evaluated direct comparison between ticagrelor monotherapy after a short course (up to 3 months) of ticagrelor-based DAPT and standard DAPT. Outcomes of interest included: (1) myocardial infarction; (2) ischemic stroke; (3) definite or probable stent thrombosis; (4) cardiovascular death; (5) all-cause death; (6) major bleeding; and (7) any bleeding.

Two reviewers (FC, MS) independently evaluated the methodological quality of the included studies using the revised Cochrane risk-of-bias tool (Rob2) assessing five domains of bias for each outcome: randomization process, deviation from intended intervention, missing outcome data, measurement of the outcome, and selection of the reported results. Disagreement was resolved with a third reviewer (GGS) [9].

For each outcome, Incidence Rate Ratios (IRRs) with 95% confidence intervals (CI) were calculated with R version 3.3.3 to facilitate a consistent interpretation of effect estimates. The Cochran’s Q test and Higgins’ I^2^ statistics were used to estimate heterogeneity among studies, with I^2^ less than 25% indicating low heterogeneity, 25–50% indicating moderate heterogeneity, and more than 50% indicating high heterogeneity. A mixed-effect Poisson regression model with random intervention effects at the study level was used to estimate the pooled IRR. A *p* value < 0.05 was considered significant. Furthermore, when data for at least two studies were available, a sensitivity analysis was performed including only studies exclusively considering anatomical definitions for complex PCI. The presence of publication bias was investigated by visual estimation of funnel plots and by the Egger test [10].

The study protocol has been registered with PROSPERO (CRD42021270744).

## 3. Results

**Figure 1:**
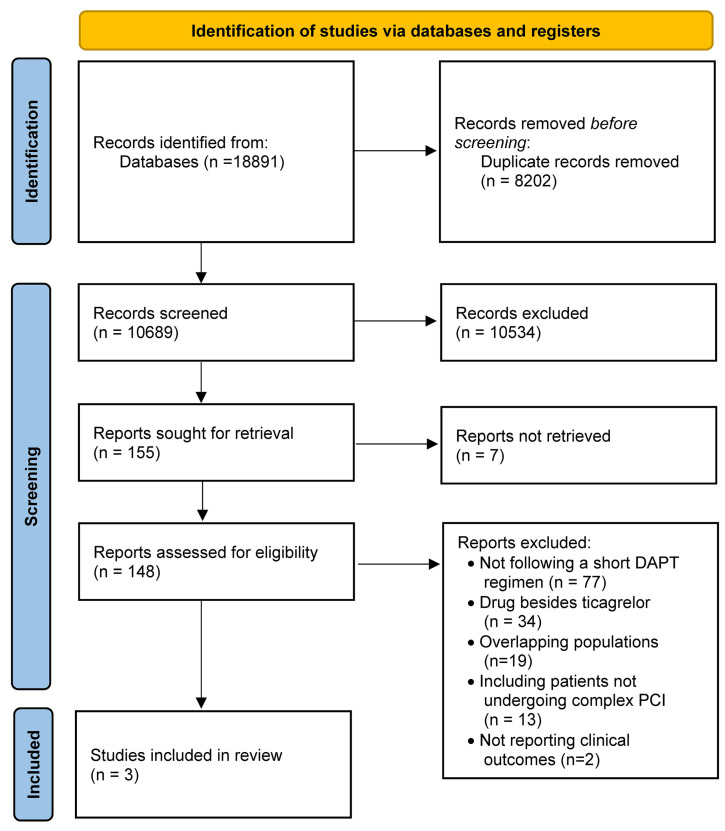
PRISMA flow chart.

The search strategy and selection process are summarized in Figure 1. Our initial search yielded 10,689 potential studies. After exclusions, three studies, including 8385 participants (4176 on ticagrelor monotherapy after a short course of ticagrelor-based DAPT; 4209 on standard DAPT), were included for analysis [11,12,13]. The main features of the included studies, baseline clinical characteristics, and the risk of bias in each study are shown in Table S1 and Figure S1. The mean age of included patients was 64.5 years, 78.4% were males, 34.6% of them suffered from diabetes, and 19.9% from chronic kidney disease. Ticagrelor-based DAPT duration was 1 month in one study [11] and 3 months in the remaining studies [12,13]. Ticagrelor monotherapy after the short course of DAPT was administered for 23 months in one study [11], 9 months in one study [13], and 12 months in one study [12]. The standard regimen was ticagrelor plus aspirin for 12 months in one study [13] and for 15 months in one study [12], while a regimen based on 12-month DAPT (aspirin and either ticagrelor for ACS or clopidogrel for stable CAD) followed by 12-month aspirin monotherapy was adopted in one study [11]. One study excluded patients presenting with ST-segment elevation myocardial infarction [12].

**Figure 2:**
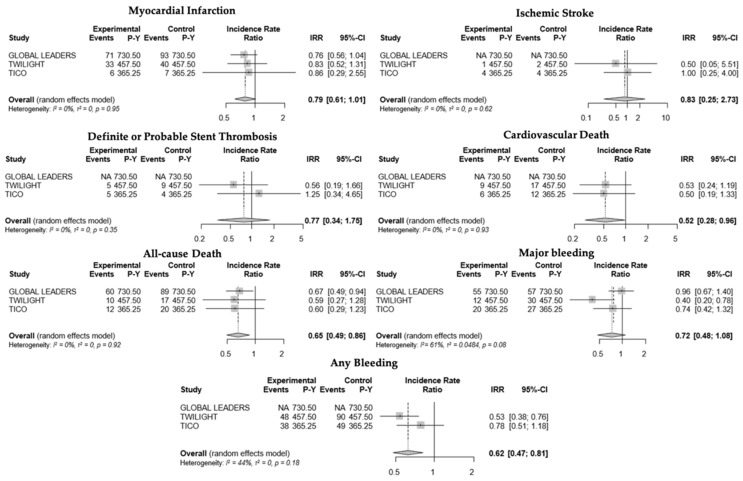
Forest plots and incidence rate ratios (IRR) with 95% confidence interval (CI) for the outcome of interest: myocardial infarction, ischemic stroke, definite or probable stent thrombosis, cardiovascular death, all-cause death, major bleeding, and any bleeding.

Overall, there was strong evidence that compared to standard treatment, ticagrelor monotherapy after a short course of ticagrelor-based DAPT (up to 3 months) reduced the risk of cardiovascular death (IRR 0.52; CI [0.28–0.96]; *p* = 0.04; I^2^ = 0%), all-cause death (IRR 0.65; CI [0.49–0.86]; *p* = 0.003; I^2^ = 0%), and any bleeding events (IRR 0.62; CI [0.47–0.81]; *p* < 0.001; I^2^ = 44%). A numerical trend between the experimental strategy and a lower risk of myocardial infarction was observed (IRR 0.79; CI [0.61–1.01]; *p* = 0.06; I^2^ = 0%). There was no significant difference in the risk of major bleeding (IRR 0.72; CI [0.48–1.08]; *p* = 0.11; I^2^ = 61%)**,** definite or probable stent thrombosis (IRR 0.77; CI [0.34–1.75]; *p* = 0.53; I^2^ = 0%), ischemic stroke (IRR 0.83; CI [0.25–2.73]; *p* = 0.76; I^2^ = 0%) between the strategy of early aspirin discontinuation after a short course of ticagrelor-based DAPT versus standard (12–15 months) DAPT (Figure 2).

Visual inspection of the funnel plots showed a slight asymmetry for the outcomes of interest; however, the Egger’s regression test was significant only for major bleeding, raising concerns about small study effect and distortion from publication bias for this outcome (Table S2; Figure S2).

The sensitivity analysis, excluding the TICO (Ticagrelor Monotherapy After 3 Months in the Patients Treated with New Generation Sirolimus-Eluting Stent for Acute Coronary Syndrome) trial, showed consistency with the main results for myocardial infarction (Table S3). A numerical trend towards a reduction in all-cause death was seen in the sensitivity analysis, while a significant reduction in major bleeding was observed.

## 4. Discussion

The main findings of the current meta-analysis can be summarized as follows: among patients undergoing complex PCI who initially completed a short course (1–3 months) of ticagrelor plus aspirin, continuation of ticagrelor monotherapy as compared to standard DAPT regimen was associated with lower incidence of any bleeding events, cardiovascular death, and all-cause death, and with comparable incidence of ischemic events. These results are in line with a previous individual patient-level meta-analysis comparing ticagrelor monotherapy after a short course of ticagrelor-based DAPT versus standard DAPT in unselected patients undergoing PCI [4]. Our analysis showed no significant difference in major bleeding events between the experimental strategy and standard therapy, while confirming the significant reduction in any bleeding events and the benefit in terms of cardiovascular death and all-cause death. We speculate that the observed mortality benefits might be related to the reduction in any bleeding events, mainly dictated by minor bleeding reduction, since major bleeding did not seem to be decreased in the shorter regimen group. The impact of minor bleeding on survival is associated with several factors: location, severity, timing, DAPT discontinuation to manage bleeding, anemia potentially leading to ischemia and greater propensity to arrhythmias due to mismatch between oxygen supply and demand, and discontinuation of other therapies in order to treat hypotension after bleeding, such as beta-blockers and renin–angiotensin–aldosterone system inhibitors, often discontinued and no longer reintroduced [14]. The TEMPLATE (Ticagrelor Monotherapy and Platelet Reactivity) trial, a study investigating the pharmacodynamic differences between ticagrelor monotherapy versus ticagrelor and aspirin in patients after PCI, demonstrated similar levels of inhibition of most platelet activation pathways with ticagrelor compared with DAPT; however, a greater aggregation response was seen with a collagen activation marker, demonstrating an incomplete inhibition of glycoprotein VI (collagen) receptor-mediated platelet activation with ticagrelor alone as compared to DAPT. This difference in response can offer a pharmacodynamic explanation in the lower bleeding rates observed in our meta-analysis and recent trials [15]. However, the observed reduction in all-cause death may have more than one explanation and non-analyzed myocardial infarction in the TICO trial may have impacted on the mortality outcome.

A subgroup analysis of the STOPDAPT-2 (Short and Optimal Duration of Dual Antiplatelet Therapy After Everolimus-Eluting Cobalt-Chromium Stent) trial showed that the beneficial effects of clopidogrel monotherapy after 1-month DAPT compared to 12-month DAPT for the primary and major secondary endpoints were comparable in complex PCI and non-complex PCI without significant interactions [16]. However, recently the STOPDAPT-2 ACS study was presented at the 2021 ESC Congress [17]. Interestingly, in patients undergoing successful PCI with DES implantation for an ACS, clopidogrel-based 1-month DAPT followed by clopidogrel monotherapy failed to achieve non-inferiority for the net combined primary endpoint of cardiovascular death, myocardial infarction, stent thrombosis, stroke, and thrombolysis in myocardial infarction (TIMI) major/minor bleeding compared to standard 12-month clopidogrel-based DAPT. There was a numerical trend towards an increase in cardiovascular events despite a reduction in major bleeding events. A possible explanation for this ischemic trend could be the low potency and high variability in the treatment response seen with clopidogrel. This is due to the metabolic activation required to generate its active metabolite via CYP2C19, which is subject to patient variability, potentially leading to high on-treatment platelet reactivity (HPR), which has been associated with a higher ischemic risk [18]. This is of particular clinical relevance in the early aftermath of ACS. The conflicting results between the two aforementioned subgroup analyses of the STOPDAPT-2 trial raise some concerns about the strategy of clopidogrel monotherapy after a short course of clopidogrel-based DAPT, at least in a subgroup of patients undergoing complex PCI. Ticagrelor is a potent P2Y12 inhibitor with more robust and less variable antiplatelet effects compared with clopidogrel. Only in rare cases do patients fail to achieve adequate platelet inhibition following ticagrelor administration. This could make physicians less concerned about recommending single antiplatelet therapy after a short course of DAPT and may lead the way towards an abbreviated course of DAPT also in the setting of complex PCI, especially in patients at high risk of bleeding [19].

Recent updates in the 2020 ESC guidelines have shed light on the application of a short DAPT strategy in patients presenting with non-ST-segment elevation ACS. The guidelines suggest considering the removal of aspirin and continuation of a P2Y12 inhibitor after 3–6 months depending on the balance between ischemic and bleeding risk (class IIa, level of evidence A) [20]. Considering the ACS subgroup in patients requiring complex PCI, each of the present studies included patients presenting with ACS: one study exclusively enrolling patients with ACS [13], one being represented in about 50% of the population [11], and one study not reporting outcome data for these patients [12]. Nevertheless, dedicated trials powered to study patients undergoing complex PCI are needed to support antiplatelet interruption in the substantial percentage of patients presenting with ACS who undergo complex PCI.

Complex PCI procedures are being increasingly performed nowadays, and more comorbid patients are being treated, therefore optimization of procedural techniques to guide clinical outcomes becomes ever more important. Often, large-bore femoral access is required, which is inherently associated with higher bleeding risks and worse clinical outcomes [21]. Nevertheless, as seen in the GLOBAL LEADERS (A Clinical Study Comparing Two Forms of Anti-Platelet Therapy After Stent Implantation) and the TICO sub-studies, radial access is increasingly used even in complex procedures: 75.6% and 53.3%, respectively, in the experimental arms (73.1% in the main TWILIGHT [Ticagrelor with Aspirin or Alone in High-Risk Patients after Coronary Intervention] study), and recent studies have suggested the superiority of the radial-first approach even in complex procedures when compared to femoral access [22]. The use of imaging techniques such as Intravascular Ultrasound (IVUS) and Optical Coherence Tomography (OCT) in procedural planning can provide assessment of plaque composition and distribution, determine the need for aggressive or less aggressive lesion preparation, and appropriate stent sizing, as well as optimizing results after stent implantation, which altogether have been shown to significantly impact clinical outcomes [23]. Overall, these considerations probably allow a safer and more impactful use of shorter DAPT duration, even in high-risk patients.

Furthermore, optimizing antiplatelet treatment through a tailored approach according to individual patient’s ischemic and bleeding risk has therefore become imperative [24]. In this respect, a great deal of help could be provided by platelet function and genetic testing in order to escalate or de-escalate DAPT to achieve the optimal HPR, which guarantees the desired level of antiplatelet effect. However, to date, relevant studies investigating a guided antiplatelet therapy and exclusively enrolling patients undergoing complex PCI are scarce. A “tailored” antiplatelet treatment using an escalation DAPT with newer P2Y12 inhibitors in patients remaining on HPR with clopidogrel and changing between prasugrel and ticagrelor in patients remaining on HPR with newer P2Y12 inhibitors was recently associated with improved clinical outcomes in a cohort of patients who underwent PCI for chronic total occlusion lesions [25]. Dedicated RCTs exclusively enrolling patients undergoing complex PCI are warranted and will clarify the role of tailored antiplatelet treatment based on platelet function and genetic testing in this group of patients. Notably, a universal definition of complex PCI procedures accepted by the entire community of interventional cardiologists is required and will facilitate future research efforts.

We acknowledge several limitations. First, the definition of “complex PCI” was heterogeneous between included studies, although most of the features were common to the complex PCI definition of each individual study. In addition, one study included clinical as well as procedural features in the definition of complex PCI [13]. Across all included trials, the definition of bleeding events varied. For instance, the TIMI bleeding definition was used in one study [13] and bleeding events according to the Bleeding Academic Research Consortium (BARC) definition were extracted from the two remaining studies, and were used for our analysis [11,12]. This may have contributed, at least in part, to the moderate–high heterogeneity observed precisely for bleeding outcomes. In the control group of the GLOBAL LEADERS trial, 51.4% of patients received a DAPT regimen of aspirin plus clopidogrel [11]. This could have led to a higher rate of ischemic events than probably would have happened with a ticagrelor-based DAPT regimen and amplified the risk difference between the two groups for ischemic events. Additionally, one trial did not report data for two of the outcomes found to be significant. Only 21.6% of participants included in the meta-analysis were women and therefore our findings cannot be directly applied to this patient population. It should also be noted that stent platform selection between studies was heterogeneous. Although, considering only second and third-generation stents were used, a negligible difference in outcomes would be expected as there are insufficient data to favor one over the other. Although two studies either excluded patients undergoing revascularization within 90 days after complex PCI (TWILIGHT) or allowed for staged revascularization within this time window (GLOBAL LEADERS), another study (TICO) did not define whether staged revascularization after PCI was allowed and whether a time frame was set for the latter not to be counted as an event. This could have impacted the duration of the short course of DAPT across each study as well as clinical outcomes. Finally, the included studies also have different time points for the evaluation of endpoints, but the mixed-effects Poisson regression model with random interventions effects allowed the aforementioned shortfall to be partially overcome.

## 5. Conclusions

In this first meta-analysis comparing ticagrelor monotherapy after a short course of ticagrelor-based DAPT and conventional DAPT in patients undergoing complex PCI with DES, ticagrelor monotherapy was associated with a lower risk of any bleeding events without compromising protection from adverse ischemic events. While the observed benefit in terms of mortality needs further investigation, the present study overall reinforces the data from recently published trials on the use of shorter-duration ticagrelor-based DAPT after complex PCI and emphasizes the need for further studies to bring about a paradigm shift in the duration of therapy among a broad spectrum of patient clinical and procedural characteristics.

## Data Availability

Data were extracted from corresponding publications of individual trials.

## Supplementary Materials

The following are available online at www.mdpi.com/article/10.3390/jcm10235506/s1, Figure S1: Risk of bias assessment by Rob2 tool for randomized controlled trials. Figure S2: Funnel plots for the pooled analysis of studies comparing ticagrelor monotherapy after a short course of dual antiplatelet therapy vs. standard dual antiplatelet therapy in complex percutaneous coronary intervention for the endpoint of myocardial infarction, ischemic stroke, stent thrombosis, cardiovascular death, all-cause death, major bleeding, and any bleeding. Table S1: Key study characteristics. Table S2: Results of the Egger test. Table S3: Sensitivity analysis excluding the TICO trial.
