# Supplementary material for: Walking the Line with Ticagrelor: Meta-Analysis Comparing the Safety and Efficacy of Ticagrelor Monotherapy after a Short Course of Ticagrelor-Based Dual Antiplatelet Therapy versus Standard Therapy in Complex Percutaneous Coronary Intervention
Source: J Clin Med. 2021 Nov 24;10(23):5506. doi: 10.3390/jcm10235506 (PMC8658113; doi:10.3390/jcm10235506)

Supplemental Material includes:

Search strategy
Figure legends for Figures from S1 to S2
Table S1 to S3
Figures S1 to S2

This supplementary material has been provided by the authors to give readers additional information about their work.

Search strategy

PUBMED
("Dual antiplatelet therapy"[tiab] OR "DAPT"[tiab] OR "P2Y12 inhibitor"[tiab] OR "ticagrelor"[tiab]) AND ("coronary artery disease"[tiab] OR "CAD"[tiab] OR "chronic ischemic heart disease"[tiab] OR "angina"[tiab] OR "acute coronary syndrome"[tiab] OR "myocardial infarction"[tiab] OR "percutaneous coronary intervention" [tiab] OR "PCI"[tiab] OR "angioplasty"[tiab] OR "drug eluting stent"[tiab] OR "DES"[tiab])

EMBASE
(('Dual antiplatelet therapy':ti,ab OR 'DAPT':ti,ab OR 'P2Y12 inhibitor':ti,ab OR 'ticagrelor':ti,ab) AND ('coronary artery disease':ti,ab OR 'CAD':ti,ab OR 'chronic ischemic heart disease':ti,ab OR 'angina':ti,ab OR 'acute coronary syndrome':ti,ab OR 'myocardial infarction':ti,ab OR 'percutaneous coronary intervention':ti,ab OR 'PCI':ti,ab OR 'angioplasty':ti,ab OR 'drug eluting stent':ti,ab OR 'DES':ti,ab))

COCHRANE
("Dual antiplatelet therapy" OR "DAPT" OR "P2Y12 inhibitor" OR "ticagrelor") AND ("coronary artery disease" OR "CAD" OR "chronic ischemic heart disease" OR "angina" OR "acute coronary syndrome" OR "myocardial infarction" OR "percutaneous coronary intervention" OR "PCI" OR "angioplasty" OR "drug eluting stent" OR "DES")

Figure legends

Figure S1: Risk of bias assessment by Rob2 tool for randomized controlled trials.

Figure S2: Funnel plots for the pooled analysis of studies comparing ticagrelor monotherapy after short dual antiplatelet therapy versus standard dual antiplatelet therapy in complex percutaneous coronary interventions for the endpoint of myocardial infarction, ischemic stroke, stent thrombosis, cardiovascular death, all-cause death, major bleeding, any bleeding

Table S1: Key study characteristics

Study	GLOBAL LEADERS		TWILIGHT		TICO	
**Country, date**	3 Continents (Europe, America, Asia), 2013-2015		3 Continents (Europe, America, Asia), 2015-2017		South Korea, 2015-2018	
**Study design**	Multicenter, randomized, open label trial		Multicenter, randomized, open label trial		Multicenter, randomized, unblended trial	
**Treatment**	23-month ticagrelor monotherapy after 1-month DAPT	12-month DAPT (aspirin and either ticagrelor for ACS or clopidogrel for stable CAD) followed by 12-month aspirin monotherapy	12-month ticagrelor monotherapy after 3-month DAPT	Ticagrelor-based 15-month DAPT	9-month ticagrelor monotherapy after 3-month DAPT	Ticagrelor-based 12-month DAPT
**No of patients**	2283	2287	1158	1184	735	738
**Population**	Patients undergoing PCI with uniform use of Biolimus A9-eluting stents and bivalirudin		Patients who underwent successful PCI (excluding STEMI) with at least 1 locally approved drug-eluting stent and in whom the treating clinician intended to discharge on regimen of ticagrelor plus aspirin		ACS patients treated with ultrathin bioresorbable polymer sirolimus-eluting stents	
**Follow up duration, months**	24		12 (i.e., 15 months after the index procedure)		12	
**Primary outcome**	Composite of all-cause death or new Q-wave myocardial infarction		BARC type 2, 3 or 5 bleeding		MACCE (composite of all-cause death, myocardial infarction, stent thrombosis, stroke, or target vessel revascularization)	
**Complex PCI**	Multivessel PCI, ≥ 3 stents implanted, ≥ 3 lesions treated, bifurcation PCI with ≥ 2 stents, total stent length > 60 mm**		3 vessels treated, ≥ 3 lesions treated, total stent length > 60 mm, bifurcation with 2 stents implanted, use of any atherectomy device, left main as target vessel, surgical bypass graft or chronic total occlusion as target lesions		Number of stents implanted ≥ 3, total stent length > 60 mm, complex procedure defined as chronic total occlusion, left main occlusion, bifurcation PCI with 2 stents, history of diabetes or chronic kidney disease	
**Definition of bleeding endpoint**	BARC		BARC		TIMI	
**Inclusion criteria**	Age ⩾18 years; patients with any clinical indication for PCI; presence of one or more coronary artery stenosis of 50% or more in a native coronary artery or in a saphenous venous or arterial bypass conduit suitable for coronary stent implantation in a vessel with a reference vessel diameter of at least 2.25 mm.		Adult patients ≥ 65 years of age; recent (≥3 days) presentation with ACS with clinical stabilization and decreasing cardiac enzymes; established vascular disease defined as previous MI, documented PAD or CAD/PAD revascularization; diabetes mellitus treated with medications (oral hypoglycemic, subcutaneous injection of insulin); CKD defined as an estimated glomerular filtration rate <60ml/min/1.73m^2 or creatinine clearance <60 ml/min; multivessel CAD; target lesion		Age ≥19 years; patients who received bioresorbable polymer sirolimus-eluting stent implantation to treat ACS; provision of informed consent	

			requiring total stent length >30 mm; SYNTAX score ≥23; bifurcation lesions with Medina X,X,1 classification requiring at least 2 stents; left main (≥50%) or proximal left anterior descending artery (≥70%) lesion; calcified target lesion requiring atherectomy.			
**Exclusion criteria**	Known intolerance to aspirin, P2Y12 receptor antagonists, bivalirudin, stainless steel or biolimus; known intake of a strong cytochrome P3A4 inhibitor (eg, ketoconazole, clarithromycin, nefazodone, ritonavir, and atazanavir), as coadministration may lead to a substantial increase in exposure to ticagrelor; use of fibrinolytic therapy within 24 hours of PCI; known severe hepatic impairment; planned CABG as a staged procedure (hybrid) within 12 months of the index procedure; planned surgery within 12 months of PCI unless DAPT is maintained throughout the peri-surgical period; need for oral anti-coagulation therapy; PCI for a priori known stent thrombosis; known overt major bleeding; known history of intracranial hemorrhage; known stroke from ischemic or unknown cause within last 30 days; known pregnancy at time of randomization; inability to provide informed consent; currently participating in another trial before reaching primary endpoint		Under 18 years of age; contraindication to aspirin; contraindication to ticagrelor; planned surgery within 90 days; planned coronary revascularization (surgical or percutaneous) within 90 days; need for chronic oral anticoagulation; prior stroke; dialysis-dependent renal failure; active bleeding or extreme-risk for major bleeding (e.g. active peptic ulcer disease, gastrointestinal pathology with a raised risk for bleeding, malignancies with a raised risk for bleeding); emergent or salvage PCI or STEMI presentation; liver cirrhosis; life expectancy < 1 year; unable or unwilling to provide informed consent; women of child bearing potential (as determined by hospital standard of care); fibrinolytic therapy within 24 hours of index PCI; concomitant therapy with a strong cytochrome P-450 3A inhibitor or inducer; platelet count < 100,000 mm3; requiring ongoing treatment with aspirin > 325 mg daily		Age >80 years; increased risk of bleeding (any prior event of hemorrhagic stroke; ischemic stroke, dementia, or impairment of central nervous system within a year; traumatic brain injury or brain surgery within the past 6 months; known intracranial tumor; documented or suspected aortic dissection; internal bleeding within the past 6 weeks; active bleeding or bleeding diathesis; anemia (hemoglobin ≤8 g/dL) or thrombocytopenia (platelet count < 100,000/μL); major surgery or traumatic injury resulting in any impairment of physical activity within the past 3 weeks); need for oral anticoagulation therapy; current or potential pregnancy; life expectancy <1 year; currently treated with strong cytochrome P4503A4 inhibitors; moderate to severe hepatic dysfunction (Child-Pugh class B or C); increased risk of bradycardia-related symptoms	
**MI**	Third Universal Definition		Third Universal Definition		Third Universal Definition after discharge	
**Stable CAD, n(%)**	1174 (51.4)	1175 (51.4)	NA	NA	0 (0)	0 (0)
**ACS, n (%)**	1109 (48.6)	1112 (48.6)	NA	NA	735 (100)	738 (100)
**Age, years, mean (SD)**	65.3 (10.3)	65.2 (10.1)	66.0 (10.4)		62.9 (10.3)	63.0 (10.6)
**Men, n (%)**	1786 (78.2)	1809 (2287)	1844 (78.7)		560 (76.2)	572 (77.5)
**Hypertension, n (%)**	1698 (74.4)	1664 (72.8)	1667 (71.2)		441 (60.0)	449 (60.8)
**Dyslipidemia, n (%)**	1545 (67.7)	1583 (69.2)	1362 (58.2)		468 (63.7)	458 (62.1)
**CKD, n (%)**	322 (14.1)	319 (13.9)	405 (18.1)		292 (39.7)	328 (44.4)
**Diabetes mellitus, n (%)**	627 (27.5)	573 (25.1)	866 (37)		418 (56.9)	417 (56.5)

ACS: acute coronary syndrome; BARC: bleeding academic research consortium; CABG: coronary artery bypass grafting; CAD: coronary artery disease; CKD: chronic kidney disease; DAPT: dual antiplatelet therapy; MACCE: major adverse cardiac and cerebrovascular events; NA: not available; PAD: peripheral artery disease; PCI: percutaneous coronary intervention; STEMI: ST-segment elevation myocardial infarction; TIMI: Thrombolysis in Myocardial Infarction

**Multivessel PCI was defined as PCI performed to treat two or three separate major coronary territories. An isolated left main lesion was classified as two-vessel disease in the presence of right dominance and three vessel disease in the presence of left dominance.

Table S2: Results of Egger test

Endpoint	P value
Myocardial Infarction	0.41
Ischemic stroke	NA
Stent thrombosis	NA
Cardiovascular death	NA
All-cause death	0.51
Major bleeding	0.0005
All bleeding	NA

NA: not available

Table S3: Sensitivity analysis excluding the TICO trial

Endpoint	IRR (95% CI)	P value
Myocardial Infarction	0.83 (0.54-1.27)	0.3890
Ischemic stroke	NA	NA
Stent thrombosis	NA	NA
Cardiovascular death	NA	NA
All-cause death	0.59 (0.35-1.01)	0.0535
Major bleeding	0.56 (0.36-0.87)	0.0090
All bleeding	NA	NA

CI: confidence interval; IRR: incidence rate ratio

Figure S1: Risk of bias assessment by Rob2 tool for randomized controlled trials

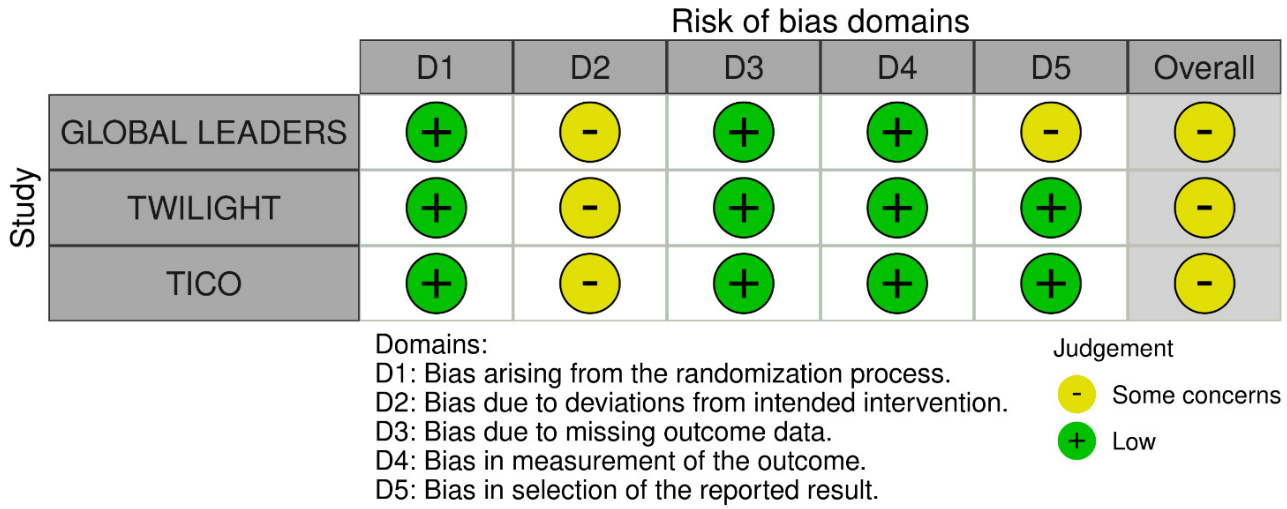

Figure S2: Funnel plots for the pooled analysis of studies comparing ticagrelor monotherapy after short dual antiplatelet therapy vs standard dual antiplatelet therapy in complex percutaneous coronary interventions for the endpoint of myocardial infarction, ischemic stroke, stent thrombosis, cardiovascular death, all-cause death, major bleeding, any bleeding.

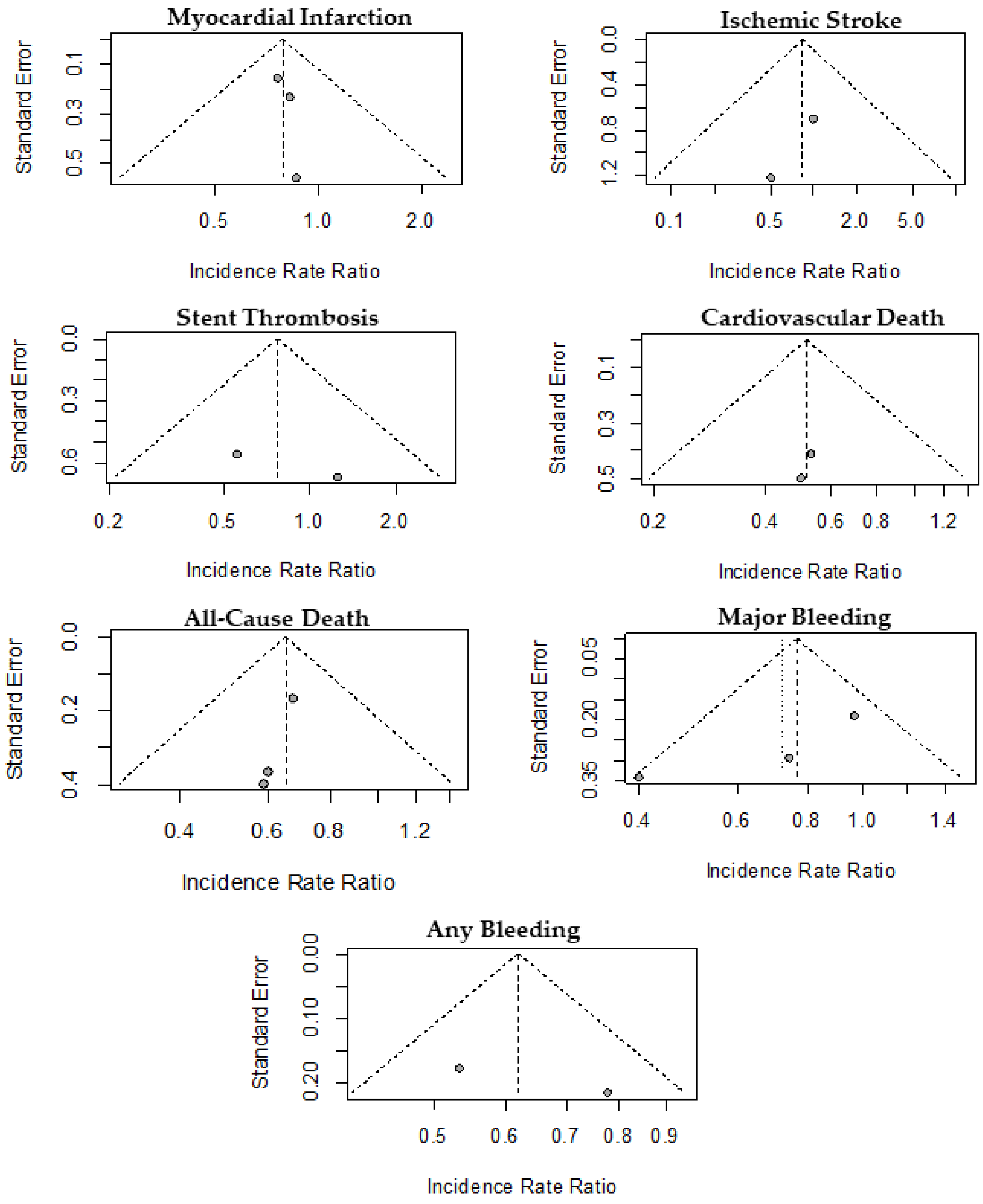

Supplement: Supplementary file 1 [file jcm-10-05506-s001.zip › jcm-1446765-supplementary.pdf]
